# Effects of alkaline or liquid-ammonia treatment on crystalline cellulose: changes in crystalline structure and effects on enzymatic digestibility

**DOI:** 10.1186/1754-6834-4-41

**Published:** 2011-10-19

**Authors:** Ashutosh Mittal, Rui Katahira, Michael E Himmel, David K Johnson

**Affiliations:** 1 Biosciences Center, National Renewable Energy Laboratory, 1617 Cole Boulevard, Golden, CO 80401, USA; 2 National Bioenergy Center, National Renewable Energy Laboratory 1617 Cole Boulevard, Golden, CO 80401, USA

## Abstract

**Background:**

In converting biomass to bioethanol, pretreatment is a key step intended to render cellulose more amenable and accessible to cellulase enzymes and thus increase glucose yields. In this study, four cellulose samples with different degrees of polymerization and crystallinity indexes were subjected to aqueous sodium hydroxide and anhydrous liquid ammonia treatments. The effects of the treatments on cellulose crystalline structure were studied, in addition to the effects on the digestibility of the celluloses by a cellulase complex.

**Results:**

From X-ray diffractograms and nuclear magnetic resonance spectra, it was revealed that treatment with liquid ammonia produced the cellulose III_I _allomorph; however, crystallinity depended on treatment conditions. Treatment at a low temperature (25°C) resulted in a less crystalline product, whereas treatment at elevated temperatures (130°C or 140°C) gave a more crystalline product. Treatment of cellulose I with aqueous sodium hydroxide (16.5 percent by weight) resulted in formation of cellulose II, but also produced a much less crystalline cellulose. The relative digestibilities of the different cellulose allomorphs were tested by exposing the treated and untreated cellulose samples to a commercial enzyme mixture (Genencor-Danisco; GC 220). The digestibility results showed that the starting cellulose I samples were the least digestible (except for corn stover cellulose, which had a high amorphous content). Treatment with sodium hydroxide produced the most digestible cellulose, followed by treatment with liquid ammonia at a low temperature. Factor analysis indicated that initial rates of digestion (up to 24 hours) were most strongly correlated with amorphous content. Correlation of allomorph type with digestibility was weak, but was strongest with cellulose conversion at later times. The cellulose III_I _samples produced at higher temperatures had comparable crystallinities to the initial cellulose I samples, but achieved higher levels of cellulose conversion, at longer digestion times.

**Conclusions:**

Earlier studies have focused on determining which cellulose allomorph is the most digestible. In this study we have found that the chemical treatments to produce different allomorphs also changed the crystallinity of the cellulose, and this had a significant effect on the digestibility of the substrate. When determining the relative digestibilities of different cellulose allomorphs it is essential to also consider the relative crystallinities of the celluloses being tested.

## Background

Cellulose is the main constituent of biomass, forming approximately 40% to 45% of the dry substance in most lignocellulosic materials and, with an estimated annual production of 10^11 ^to 10^12 ^tons, it is the world's most abundant biological material [1-3]. Cellulose is a linear, unbranched homopolysaccharide composed of β-D-glucopyranose units which are linked together by β-1,4-glycosidic bonds to form a crystalline material. The cellulose degree of polymerization (DP) ranges from 500 to 15,000. The basic building unit of the cellulose skeleton is an elementary fibril, which is formed from insoluble microfibrils. The microfibrils are oriented along the longitudinal axis of the fibrils and are considered to be a bundle of 36 parallel cellodextrin chains which are held together by intermolecular (interchain and intrachain) hydrogen bonds [4,5]. Cellulose contains crystalline and amorphous regions, and crystallinity, a measure of the weight fraction of the crystalline regions [6], is one of the most important measurable properties of cellulose that influences its enzymatic digestibility [7-9]. Many studies have shown that completely disordered or amorphous cellulose is hydrolyzed at a much faster rate than partially crystalline cellulose [7-9], which supports the idea that the initial degree of crystallinity is important in determining the enzymatic digestibility of a cellulose sample.

Four different crystalline allomorphs of cellulose (cellulose I, II, III and IV) have been identified by their characteristic X-ray diffraction patterns and solid-state ^13^C nuclear magnetic resonance (NMR) spectra. Cellulose I is the most abundant form found in nature. It is known that the crystalline structure of cellulose I is a mixture of two distinct crystalline forms, cellulose I_α _(triclinic) and cellulose I_β _(monoclinic) [10]. Cellulose I_α _is the predominant form found in bacteria and algae, whereas the cellulose in higher plants is mostly I_β_. Cellulose II can be prepared by two distinct routes, mercerization (alkali treatment) and regeneration (solubilization and subsequent recrystallization). Cellulose III_I _and III_II _can be formed from cellulose I and II, respectively, by treatment with liquid ammonia; the reaction is, however, reversible [11]. Cellulose IV_I _and IV_II _can be obtained by heating cellulose III_I _and III_II_, respectively [12]. A thorough review of cellulose crystalline allomorphs can be found elsewhere [13-15].

In the conversion of biomass to bioethanol, pretreatment of biomass is a key step intended to render cellulose more amenable and accessible to cellulase enzymes, thereby increasing glucose yields. Due to its rigid structure and crystallinity, cellulose provides the basic framework for plant fibers and is resistant to enzymatic hydrolysis. The enzymatic hydrolysis of cellulose is a slow process and the extent of hydrolysis is influenced by the structural properties of the biomass substrate, such as cellulose crystallinity, surface area, degree of polymerization and porosity [8,16]. In order to increase the efficiency and efficacy of the enzymatic hydrolysis process, it is necessary to perform a chemical pretreatment of the biomass to modify its composite structure, thereby allowing better access to cellulose by cellulase enzymes. Some chemical treatments, such as with liquid ammonia and aqueous sodium hydroxide (NaOH), are known to alter the crystalline structure of cellulose, resulting in the formation of different allomorphs that have different unit cell dimensions, chain packing schemes and hydrogen bonding relationships [17-20].

Liquid ammonia treatment of cellulose has been studied for many years [21-23]. Ammonia is believed to swell cellulose and penetrate its crystal lattice to yield a cellulose-ammonia complex [21,24]. According to Lewin and Roldan [25], during liquid ammonia treatment the ammonia molecules penetrate cellulose and react with the hydroxyl groups after breaking the hydrogen bonds. The interaction between cellulose and liquid ammonia [21] or primary amines [26] causes changes in interplanar distances of the 101 planes, resulting in a unit cell with increased volume from 671 cubic Å for native cellulose I to 801 cubic Å for the cellulose-ammonia complex. As the ammonia is allowed to evaporate, the volume of a unit cell of dried ammonia-treated cellulose decreases to 702 cubic Å [25]. The volume of the unit cell of cellulose III_I _thus formed is less than that of the cellulose-ammonia complex, but is slightly larger than that of native cellulose I. It has been postulated, therefore, that cellulose III_I _should have enhanced cellulase accessibility and chemical reactivity [18]. The degree of conversion of cellulose I to cellulose III_I _depends on various operational parameters [27], such as the reaction period and the temperature used in the final stage of the treatment [25].

Recently, Igarashi *et al*. [28] reported that supercritical ammonia treatment of crystalline cellulose I produced an activated form of cellulose that exhibited enhanced hydrolysis by cellobiohydrolase I (Cel7A), producing cellobiose at rates more than five times higher than from cellulose I. These authors concluded that supercritical ammonia treatment activated crystalline cellulose for hydrolysis by Cel7A; however, this study was only performed on the algal cellulose from *Cladophora*, which consists predominately of the I_α _allomorph.

Another chemical treatment that has been used extensively to alter the molecular and supramolecular cellulose structure is treatment with 8% to 25% aqueous NaOH solution [1,29]. The treatment of cotton linters (cellulose I) with 12% to 18% NaOH solution has been reported to convert cellulose I to cellulose II with decreased crystallinity [29,30].

The aqueous NaOH and anhydrous liquid ammonia treatments alter the crystalline structure of cellulose, resulting in the formation of different allomorphs, in other words, cellulose II and cellulose III_I_, respectively. However, besides producing the different allomorphs of cellulose, these chemical treatments can also alter other physical properties of cellulose, such as the degree of crystallinity, a property considered to play a major role in the enzymatic digestibility of cellulose [8].

The purpose of this study was to investigate the changes in cellulose crystal structure caused by treatment of different cellulose samples with aqueous NaOH or anhydrous liquid ammonia. Cellulose allomorphs were prepared from four cellulose samples with differing physical properties. The treated celluloses were characterized in detail and the impact of changes in their properties was correlated with the enzymatic digestibilities of the samples.

## Results

### Liquid ammonia treatments

**Figure 1:**
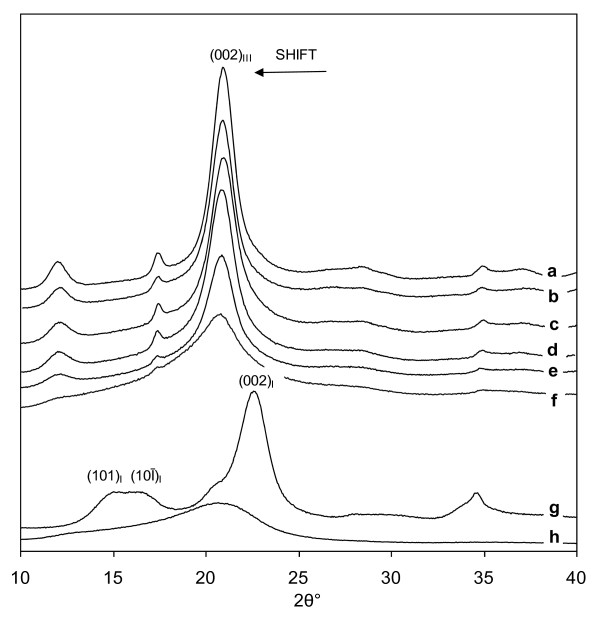
**X-ray diffractograms showing the effect of temperature and hold time during liquid ammonia treatment on conversion of cellulose I to cellulose III**_**I**_. (a) 140°C, (b) 130°C, (c) 105°C, (d) 25°C for 90 min, (e) 25°C for 5 min, and (f) -33°C for 15 min, (g) the Avicel PH 101 control cellulose I, (h) is amorphous cellulose.

Figure 1 shows the X-ray diffractograms for Avicel PH 101 treated with liquid ammonia at different temperatures and hold times. The change from cellulose I to cellulose III_I _can be observed by following the position of the 002 peak, which shifts from a 2θ value of 23 to 21 as cellulose I is converted to cellulose III_I_. The diffractograms in Figure 1 show that under all conditions cellulose I was completely converted to cellulose III_I_, however the treatments also affected the crystallinity of the cellulose III_I _product. It is well known that, in X-ray diffractograms, amorphous solids with small particle size and lattice distortion show broad maxima, whereas crystalline solids have sharper peaks indicating larger crystallites [31-33]. From (a) to (d), the effect of removing ammonia at higher temperatures can be clearly observed, as the increased peak height and decreased peak width of the 002 peak indicates a decrease in the disordered (amorphous) fraction [34]. The sample heated to 140°C (Figure 1a) contained cellulose III_I _with high crystallinity, characterized by a narrow 002 peak with high intensity, whereas the sample treated at -33°C (Figure 1f) contained cellulose III_I _that was more amorphous and so had a much broader 002 peak with lower intensity.

Figure 1(d) and 1(e) show the effect of hold time at a final temperature of 25°C on the crystallinity of the cellulose III_I _product. Comparing (d) to (e), it would appear that increasing the hold time to 90 min resulted in a more crystalline cellulose III_I _product.

Data on the degree of crystallinity, amorphous content and crystallite size, for Avicel PH 101 treated with liquid ammonia under different conditions is given in Table 1. It can be noted that increasing the final temperature to which the cellulose was heated during ammonia treatment resulted in cellulose III_I _samples with higher crystallinity index (CI). For example, liquid ammonia treatment at a final temperature of 140°C for 1 h produced cellulose III_I _sample (a) with a CI of 78%, which was actually higher than the CI of the starting cellulose I (74%). When the reactor was depressurized at -33°C, allowing the ammonia to boil off before the cellulose was exposed to ambient temperature, cellulose III_I _sample (f) had a very low degree of crystallinity (43%) and an amorphous fraction of almost twice that of the starting cellulose I.

**Table 1:** Crystalline (cellulose I or cellulose III_I_) and amorphous cellulose contents (measured by XRD) of samples prepared from Avicel PH 101 after ammonia treatment at different final temperatures

Sample number - Figure 2	**NH**_**3 **_**treatment conditions**	Amorphous content (%)	Cellulose allomorphs (CI, %)		Crystallite size (Å)
				
			I	III_I_	
(g)	Control^a^	26	74 (1.2)	-	46 (1.1)
(f)	-33°C	57	-	43	20
(e)	25°C^b^	46	-	54	38
(d)	25°C	36	-	64	42
(c)	105°C	35	-	65	49
(b)	130°C	26	-	74	55
(a)	140°C	22	-	78	57

^a^Values for control (Avicel PH-101) were measured three times and the numbers in parentheses indicate the standard deviation; ^b^NH_3 _treatment at 25°C for 5 min. CI: crystallinity index; XRD: X-ray diffraction.

**Figure 2:**
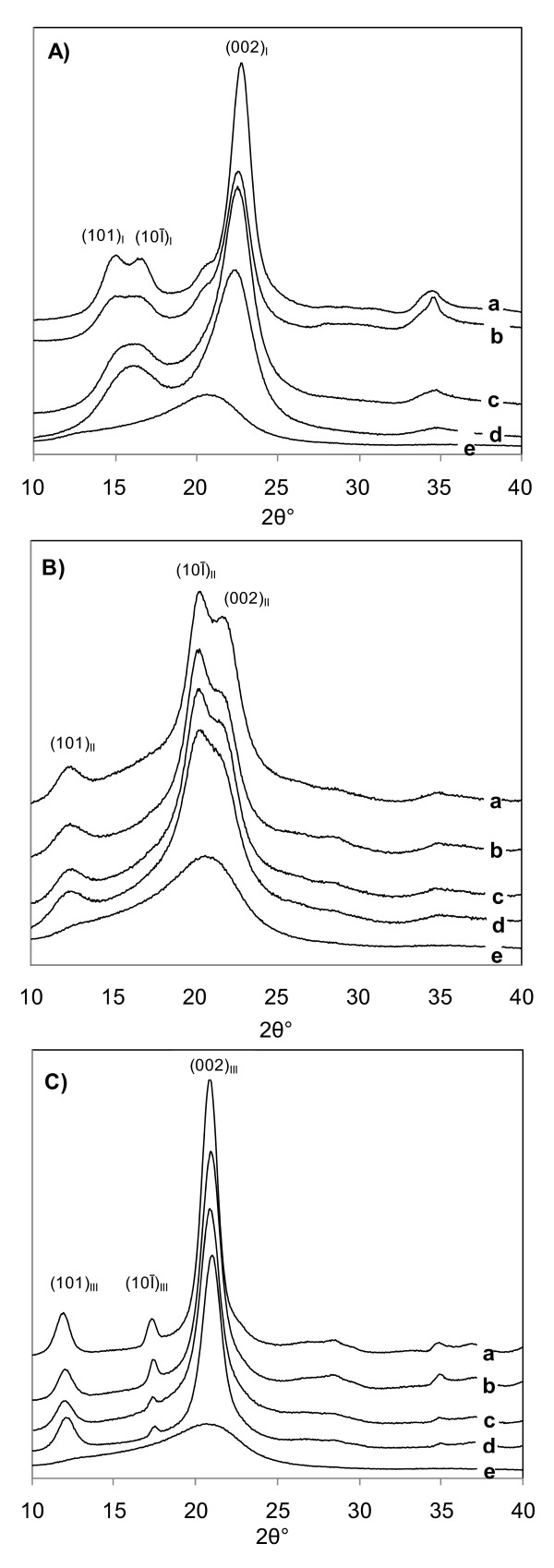
**X-ray diffractograms**. **(A) **untreated cellulose I, **(B) **cellulose I treated with NaOH to make cellulose II, **(C) **cellulose I treated with ammonia at 130°C to make cellulose III_I_, from different cellulose samples. (a) cotton linters, (b) Avicel PH 101, (c) α-cellulose, (d) cellulose isolated from corn stover, (e) amorphous cellulose.

Based on the results of preparing cellulose III_I _samples from Avicel, two liquid ammonia treatment conditions were selected for the treatment of three other celluloses to produce cellulose III_I _samples. These samples were used to study the effect of crystalline structure on enzymatic digestibility. The conditions selected were treatment at a final temperature of 130°C for 1 h and at 25°C for 5 min. In the literature [27], production of highly crystalline cellulose III_I _was obtained by supercritical ammonia treatment at 140°C; however, in our hands, a darkly colored product was obtained at this temperature that had a lower glucan content, indicating that degradation of the cellulose had occurred. In order to minimize cellulose degradation during liquid ammonia treatment at higher temperatures, a final temperature of 130°C was selected as this yielded a cellulose III_I _sample with off-white color, little apparent glucan degradation (Table 2), and crystallinity comparable to that of the starting cellulose I. X-ray diffraction (XRD) of the cellulose III_I _samples made from different celluloses are shown in Figure 2C.

**Table 2:** Glucan content of cellulose samples obtained after NaOH treatment at 25°C and liquid ammonia treatment at final temperatures of 25°C and 130°C

Glucan content (%)	Untreated (control)	**NH**_**3 **_**treated**		NaOH treated
			
		25°C	130°C	
Avicel	95 ± 1.0	96.5 ± 2.0	94 ± 1.2	97 ± 1.1
α-Cellulose^a^	92 ± 0.5	93 ± 0.7	92 ± 0.9	95 ± 0.4
Cotton linters	99 ± 0.7	98 ± 0.6	99 ± 0.5	100 ± 0.6
Corn stover^b^	83 ± 1.1	83 ± 0.8	83 ± 1.3	84 ± 1.2

^a^Xylan 2.3%, Klason lignin 0.9%; ^b^Xylan 5.3%, Klason lignin 3.1%.

The other liquid ammonia treatment condition selected was 25°C for 5 min, as this was relatively easily obtained and gave cellulose III_I _with a low crystallinity. Ammonia treatment at two different conditions resulted in formation of cellulose III_I _samples with contrasting crystallinities and crystallite sizes. As can be seen in Table 3, for all four cellulose samples ammonia treatment at a final temperature of 25°C for 5 min resulted in an increase in the amorphous fraction; however, there was no significant reduction in the crystallite size of these cellulose samples. Interestingly, liquid ammonia treatment at a final temperature at 130°C for 60 min resulted in the formation of cellulose III_I _with a higher CI and much larger crystallite sizes than even the starting cellulose I for all cellulose samples.

**Table 3:** Crystalline and amorphous cellulose contents (measured by XRD) of samples obtained using various ammonia treatment conditions and aqueous NaOH treatment

Cellulose samples	Treatment conditions		Amorphous content (%)	Cellulose allomorphs (CI, %)			Crystallite size (Å)	Cellulose conversion (%)	
						
				I	II	III_I_		16 h	24 h
Avicel	Control		26	74	-	-	46	39	46
	
	NaOH	25°C	47	-	53	-	37	66	76
	
	NH_3_	25°C	46	-	-	54	38	49	59
		
		130°C	26	-	-	74	55	36	49

α-cellulose	Control		42	58	-	-	40	41	48
	
	NaOH	25°C	51	-	49	-	33	65	77
	
	NH_3_	25°C	45	-	-	55	45	51	62
		
		130°C	36	-	-	64	55	43	56

Cotton linters	Control		27	73	-	-	55	36	41
	
	NaOH	25°C	56	-	44	-	40	67	78
	
	NH_3_	25°C	48	-	-	52	57	43	52
		
		130°C	24	-	-	76	68	42	53

Corn stover	Control		50	50	-	-	30	78	86
	
	NaOH	25°C	56	-	44	-	34	79	87
	
	NH_3_	25°C	49	-	-	51	54	76	85
		
		130°C	32	-	-	68	56	55	67

CI: crystallinity index; XRD: X-ray diffraction.

### Aqueous NaOH treatments

Alkaline treatments were performed on cellulose I samples using 16.5 weight percent NaOH for 2 h at 25°C to convert them into the cellulose II allomorph. The change from cellulose I to cellulose II is denoted by the appearance of a doublet in the XRD for the 10Ī and 002 peaks (at 2θ values of about 20 and 22) as shown in Figure 2B. Generally, the aqueous NaOH treatment of all four cellulose samples caused a substantial reduction in both cellulose crystallinity and crystallite size. Treatment of the two most crystalline cellulose samples, Avicel and cotton linters, with NaOH resulted in two-fold increases in the amorphous fraction. This finding is consistent with results reported in the literature [29,30].

### XRD analyses

Figure 2 shows the XRD of the three allomorphs for the four different cellulose samples; (a) cotton linters, (b) Avicel PH 101, (c) α-cellulose, (d) corn stover. The XRD of the three allomorphs are essentially the same as XRD reported in the literature [25,29,32,34]. Data on the degree of crystallinity, amorphous content and crystallite size (as measured by XRD) for all untreated and treated samples are reported in Table 3.

### ^13^C cross polarization and magic angle spinning solid-state NMR analyses

**Figure 3:**
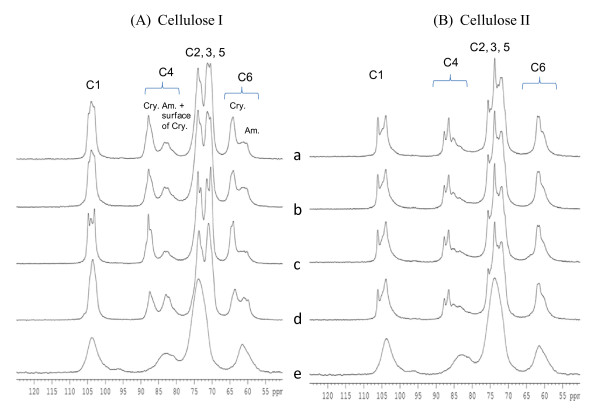
**^13^C CPMAS solid-state NMR spectra**. **(A) **Original cellulose I and **(B) **cellulose II for various cellulose samples. (a) Avicel, (b) α-cellulose, (c) cotton linters, and (d) corn stover, (e) amorphous cellulose. CPMAS: cross polarization and magic angle spinning solid-state; NMR: nuclear magnetic resonance.

The ^13^C cross polarization and magic angle spinning solid-state (CPMAS) NMR spectra of cellulose I and II from Avicel PH 101, α-Cellulose, IFC cotton linters and corn stover cellulose are shown in Figure 3A and 3B, respectively. All peaks were assigned by comparison with literature spectra [35] and CI values were calculated for each cellulose allomorph (Table 4). Each spectrum of the cellulose I samples had distinct C4 crystalline and amorphous peaks. Avicel PH101, α-cellulose and corn stover celluloses have similar spectra; however, the peaks in the spectrum from cotton linter cellulose were sharper and narrower. This is due to the high crystallinity and high DP of the cellulose in cotton linters. In the NMR spectra of the aqueous NaOH treated celluloses (Figure 3B), the shape of each peak was changed, especially for the signals assigned to the C4 carbon; the crystalline and amorphous peaks in the C4 region (80 ppm to 90 ppm) were shifted. Due to decreased separation between the peaks, the method for calculating the CI of cellulose II samples by spectral subtraction is probably less accurate. However, the NMR spectra also indicate that the CI values for the cellulose II samples were lower than for the original cellulose I samples.

**Table 4:** Comparison of crystallinity index measured by XRD and NMR for cellulose samples obtained using aqueous NaOH and liquid ammonia treatments

Cellulose samples	Treatment conditions		Crystallinity index (%)	
			
			XRD	NMR
Avicel	Control		74	84
	
	NaOH	25°C	53	74
	
	NH_3_	25°C	54	54
		
		130°C	74	67

α-cellulose	Control		58	66
	
	NaOH	25°C	49	66
	
	NH_3_	25°C	55	53
		
		130°C	64	63

Cotton linters	Control		73	82
	
	NaOH	25°C	44	66
	
	NH_3_	25°C	52	62
		
		130°C	76	75

Corn stover	Control		50	65
	
	NaOH	25°C	44	60
	
	NH_3_	25°C	51	50
		
		130°C	68	69

NMR: nuclear magnetic resonance; XRD: X-ray diffraction.

**Figure 4:**
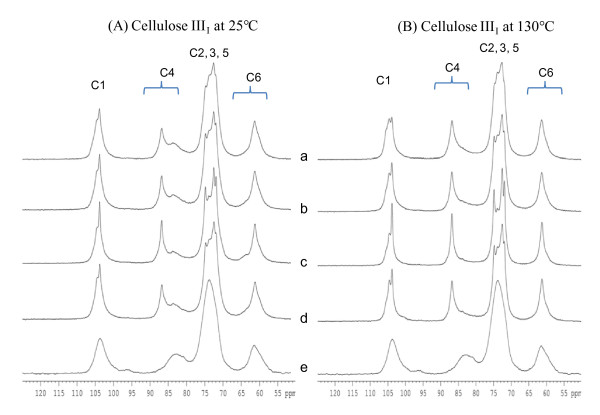
^**13**^**C CPMAS solid-state NMR spectra of cellulose III**_**I **_**prepared by treatment with liquid ammonia**. **(A) **At 25°C and **(B) **130°C from various cellulose I samples. (a) Avicel, (b) α-cellulose, (c) cotton linters, and (d) corn stover, (e) amorphous cellulose. CPMAS: cross polarization and magic angle spinning solid-state; NMR: nuclear magnetic resonance.

The NMR spectra of cellulose III_I _obtained after liquid ammonia treatment at 25°C and 130°C are shown in Figure 4A and 4B, respectively. All peaks were assigned by comparison with literature spectra [36] and CI values were calculated (Table 4). The spectra of cellulose III_I _prepared at 25°C (Figure 4A) from α-cellulose and corn stover cellulose appear similar. The cotton linters spectrum has sharper peaks in all regions compared to the spectra of the other celluloses, probably because cotton linters cellulose has a higher molecular weight and a more crystalline structure (Table 5). The spectra of cellulose III_I _prepared at 130°C show higher C4 crystalline peaks and much lower C4 amorphous peaks (Figure 4B), indicating that the CI of each cellulose III_I _sample increased at 130°C (Tables 3 and 4). The NMR results confirm that the CI of the cellulose III_I _varied with the treatment conditions. Comparing the CI values obtained by XRD to those obtained from the NMR spectra (Table 4), it appears that the NMR gives higher CI values for cellulose I and II samples, but very similar CI values for the cellulose III_I _samples.

**Table 5:** The degree of polymerization and crystallinity of the initial cellulose samples used in this study

Cellulose samples	**DP**_**n**_	**DP**_**w**_	**CI**^**a**^
Avicel PH 101	90	480	74
α-Cellulose	160	2000	57
Cotton linters	600	6600	73
Corn stover	170	3100	50

^a^CI was measured by applying the amorphous subtraction method to the XRD of the celluloses. CI: crystallinity index; DP: degree of polymerization; XRD: X-ray diffraction.

### Cellulose DP analyses

**Figure 5:**
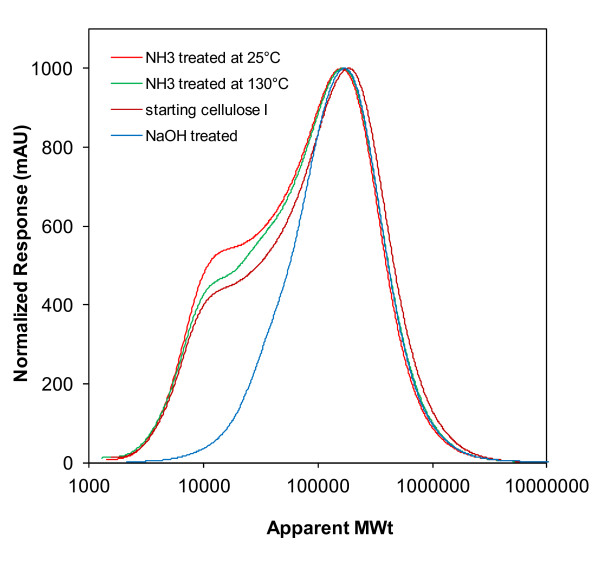
**Molecular weight distributions of Avicel PH 101 and treated Avicel samples**.

Some studies have shown that cellulose digestibility increases as DP decreases [37], whereas Sinitsyn and colleagues [38] reported no correlation between DP and digestibility. In this work, the liquid ammonia treatments caused only small changes in cellulose DP (Figure 5). NaOH treatment resulted in solubilization of the lowest molecular weight fraction (< 40 DP). This had a greater effect on the lowest DP cellulose, Avicel, resulting in removal of about 20% of the cellulose, compared with almost no effect on the highest DP cellulose, cotton linters, which had almost no material in this low molecular weight range. It is unlikely that these small changes in cellulose DP were the cause of the large differences in digestion rates for the cellulose samples treated with NaOH or anhydrous ammonia.

### Enzymatic digestions

**Figure 6:**
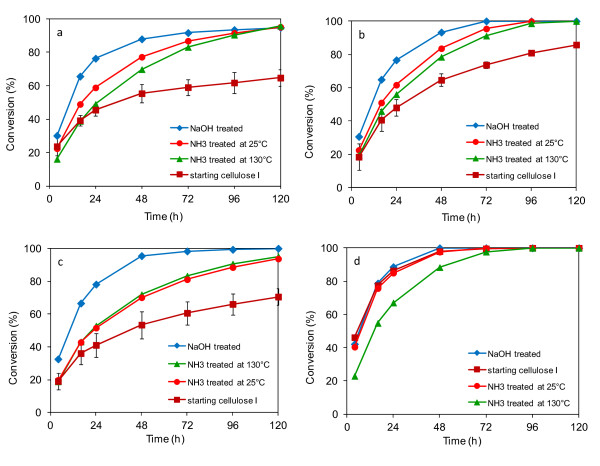
**Enzymatic hydrolysis of different cellulose samples**. (a) Avicel PH 101, (b) α-cellulose, (c) cotton linters, (d) corn stover cellulose. (The error bars indicate the reproducibility (± 1 standard deviation) of digestions conducted in triplicate on the Avicel cellulose samples).

All treated and untreated cellulose samples were enzymatically digested in shake flasks in duplicate (Figure 6). The variability in the digestions was about 3%, calculated from triplicate digestions performed on Avicel. From Figure 6, it can be seen that the maximum cellulose hydrolysis rates were obtained for the NaOH treated cellulose samples. Generally, the NaOH and ammonia treated celluloses had higher enzymatic digestion rates than the starting cellulose I samples. The initial hydrolysis rates (digestion period from 4 to 24 h) for the treated celluloses were about two times faster compared to the starting cellulose I for all three of the commercial celluloses. The initial hydrolysis rates and maximum cellulose conversions to total glucose plus cellobiose for the three cellulose treatments were found to be greatest with NaOH, followed by ammonia at 25°C, then ammonia at 130°C cellulose, with the lowest rates for the initial cellulose I. This was true for all but the corn stover cellulose. This cellulose was distinguished from the other celluloses by its low CI and the consequently very high digestibility of the original cellulose I, which was almost as digestible as the NaOH and low temperature ammonia treated samples. For the corn stover cellulose, the high temperature ammonia treated sample was the most crystalline and the least digestible.

## Discussion

### Effect of ammonia treatment on cellulose crystalline structure

It has been reported in the literature that temperature and duration of treatment with liquid ammonia are critical in determining the crystal lattice and degree of crystallinity [25,27,39]. In this study, after ammonia treatment complete conversion of cellulose I to cellulose III_I _was observed with no remnant of cellulose I in the treated cellulose samples. However, the CI of the cellulose III_I _varied with the treatment conditions. According to the mechanism of liquid ammonia treatment by Schuerch [24], the anhydrous ammonia penetrates both the amorphous and crystalline regions in cellulose by breaking hydrogen bonds, resulting in the formation of an ammonia-cellulose complex. Various researchers [21,40] have reported that the formation of the ammonia-cellulose complex results in a larger unit cell than native cellulose. As ammonia is allowed to evaporate, new hydrogen bonds are formed which results in a completely new pattern of hydrogen bond cross-linking [24]. This generates the unit cell of cellulose III_I _[40].

Lewin and Roldan [25] produced a phase diagram for the treatment of cellulose with ammonia that involved cellulose I, the ammonia-cellulose complex, disordered cellulose and cellulose III_I _. The transition from cellulose I to the ammonia-cellulose complex was obtained by application of liquid ammonia. The transition from the ammonia-cellulose complex to disordered cellulose or cellulose III_I _was possible depending on whether the treatment and ammonia removal were performed in the absence or presence of heat, and cellulose I could be reformed by the application of heat and water. The crystallinity of the cellulose III_I _has been reported to depend on the temperature of treatment and removal of ammonia. Yatsu and coworkers [27] reportedly obtained highly crystalline cellulose III_I _by supercritical ammonia treatment of cotton at 140°C, whereas Saafan and coworkers [39] reported that a highly disordered material was obtained by treating cotton with liquid ammonia at -33°C for 1 min. In our experiments with Avicel cellulose, we examined conditions that achieved transformations of the ammonia-cellulose complex over a wide range of ratios of disordered cellulose to cellulose III_I_. It is clear that treatment time, temperature, and the samples' moisture contents can be manipulated to obtain ammonia treated samples with almost all levels of amorphous content, from highly disordered to highly ordered cellulose III_I _samples; even to the extent where the crystallinity of the product is greater than that of the starting cellulose. A few preliminary experiments have also been conducted that converted completely amorphous cellulose into cellulose exhibiting a significant degree of order.

### Effect of crystalline structure on enzymatic digestibility

The interactions between properties such as crystalline structure, degree of polymerization, accessible surface area and particle size are complex. Their effects on cellulose digestibility are also difficult to assess. During pretreatment, a change in one property is often accompanied by changes in other properties [8,20,41,42]. This work has tried to distinguish the effect of changes in cellulose crystalline structure from changes in the other properties that occur during treatment of biomass with aqueous NaOH or anhydrous liquid ammonia. These treatments produced samples whose XRD indicated there had been changes in their crystalline structures that have been assigned by other researchers to cellulose allomorphs II [29] and III_I _[18]. However, it is evident from the amorphous content data (Table 3) that these treatments also caused significant changes in crystallinity and as much as a two-fold increase in the amorphous content of the celluloses, thus making it difficult to assess the effect of changing the allomorph from changes in crystallinity on the digestibility of the samples.

The relative digestibilities of cellulose allomorphs have been investigated in previous studies on the two designated crystalline forms of cellulose I [10], cellulose I_α _[28] and I_β _[20]. Weimer and coworkers [20] compared the digestibility of amorphous cellulose and all four crystalline allomorphs (I, II, III, and IV) of cellulose prepared from cotton fiber (predominantly cellulose I_β_) digested by ruminal cellulolytic bacteria. In their work, Weimer and coworkers reported that the initial hydrolysis rates for different allomorphs digested with *R. flavefaciens *to be in the following order: amorphous > III_I _> IV_I _> III_II _> I > II. They attributed the highest digestibility rates (approximately 10-fold compared to the starting cellulose I) obtained for amorphous cellulose to its four-fold higher gross specific surface area compared to the starting cellulose I. Additionally, the enhanced digestibility rate of III_I _was attributed to its reduced crystallinity and the difference in the lattice structures of cellulose I and III_I_. In recent work, Igarashi and coworkers [28] reported on the enzymatic digestibility of different crystalline allomorphs of *Cladophora *cellulose (algal cellulose - predominantly cellulose I_α_) by *T. reesei *Cel7A to be in the following order: cellulose III_I _> cellulose I_α _> cellulose I_β_. They also stated that the enhanced digestibility rate of III_I _was due to its reduced cellulose crystallinity and the difference in crystal structure of cellulose I and III_I_, which is believed to have a lower packing density and greater distances between hydrophobic surfaces than cellulose I. In this work we have tried to assess the relative importance of changing crystallinity to changing allomorph type in determining the digestibility of cellulose samples.

In Figure 6, the very different digestibilities of the starting cellulose I samples compared to the treated celluloses can be seen. The levels of conversion of untreated Avicel, cotton linters and, to a lesser extent, α-cellulose were much lower than the treated samples. This was not the case for the corn stover cellulose I sample. As can be seen in Table 3, Avicel and cotton linters, among the starting cellulose samples, had the lowest amorphous contents (26% and 27%, respectively); whereas, α-cellulose and corn stover cellulose had high amorphous contents (42% and 50%, respectively). Although the higher amorphous contents of the α-cellulose and corn stover cellulose may have been at least partially due to their lower cellulose contents due to the presence of xylan and lignin in the samples (Table 2), these data nevertheless indicate the importance of crystallinity in determining cellulose digestibility.

It is apparent that the NaOH treated celluloses were all very digestible; although NaOH treatment produced the cellulose II allomorph it also resulted in a much higher level of amorphous cellulose than the other celluloses tested (Table 3). The enhanced enzymatic digestibility of NaOH treated celluloses over that of the untreated celluloses and ammonia treated cellulose samples is likely due to the significant increase in the disordered or amorphous fraction in the NaOH treated cellulose samples. It is also important to note from Table 3 that there was a significant reduction in crystallite size for the NaOH treated cellulose samples.

The samples obtained by treatment with ammonia at a final temperature of 25°C were also relatively digestible, although not quite as digestible as the NaOH treated celluloses, and their amorphous contents were mostly lower than the NaOH treated celluloses (Table 3). The low temperature ammonia treatment resulted in samples containing the cellulose III_I _allomorph, but also a higher level of amorphous cellulose compared to the starting celluloses. The digestibility of the low temperature ammonia treated celluloses was less than the NaOH treated celluloses and higher than the starting celluloses.

The samples obtained by treatment with ammonia with a final temperature of 130°C were less digestible than the samples treated at 25°C (except for the cotton linter samples) and had lower levels of amorphous cellulose compared to the starting celluloses. In the case of samples made from cotton linters, the digestibility of the 130°C treated cellulose was the same as the lower temperature treatment despite their lower amorphous contents (24% versus 48%). The corn stover cellulose treated at 130°C was much less digestible than all of the other samples obtained from corn stover, which all had higher amorphous contents (approximately 50% versus 32%). In assessing the high temperature ammonia treated samples, it is clear that they are generally less digestible than the low temperature ammonia treated and NaOH treated samples. In three of these cases, the high temperature ammonia treated samples were more digestible than the starting celluloses.

**Figure 7:**
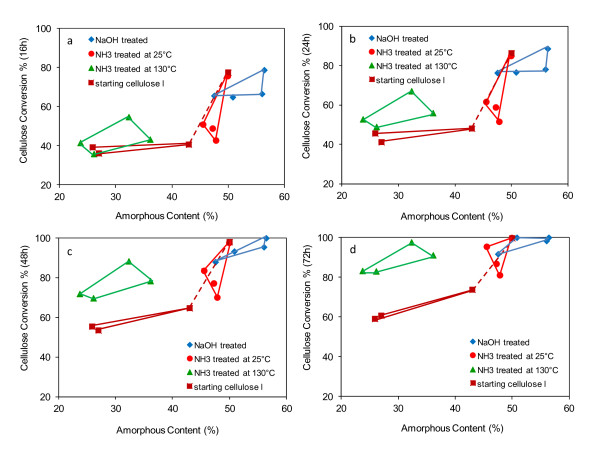
**Comparison of cellulose conversion levels after (a) 16 h, (b) 24 h, (c) 48 h, and (d) 72 h for the starting cellulose I, NaOH treated, NH**_**3 **_**treated at 25°C, and NH**_**3 **_**treated at 130°C**. (Note: The highly digestible, high amorphous content, corn stover cellulose I sample is connected to the other cellulose I samples by a dashed line).

In Figure 7, the effect of amorphous content on the digestibility of the cellulose samples is presented. It appears that the samples with higher amorphous content were more rapidly digested than the more crystalline samples, especially early in the digestions. NaOH treated samples had the highest amorphous contents and the highest cellulose conversions, whereas the untreated samples with lower amorphous contents had the lowest conversions. Both the corn stover cellulose treated with ammonia at 25°C and the untreated corn stover cellulose had high amorphous contents and appeared as digestible as the NaOH treated samples, adding further support for the idea that amorphous content has a strong influence on digestibility.

The samples treated with ammonia at 130°C behaved somewhat differently to the other samples in that they had low levels of conversion early in the digestions, similar to the untreated celluloses that had similar high crystallinities. At later times in the digestions, however, they achieved much higher cellulose conversions than the more crystalline untreated celluloses (Figure 7) This appears to be evidence that the cellulose III_I _allomorph is more digestible than the cellulose I allomorph when the CI of the celluloses are similar. The higher digestibility of the cellulose III_I _allomorph is in agreement with earlier findings that this allomorph is more digestible than cellulose I.

**Figure 8:**
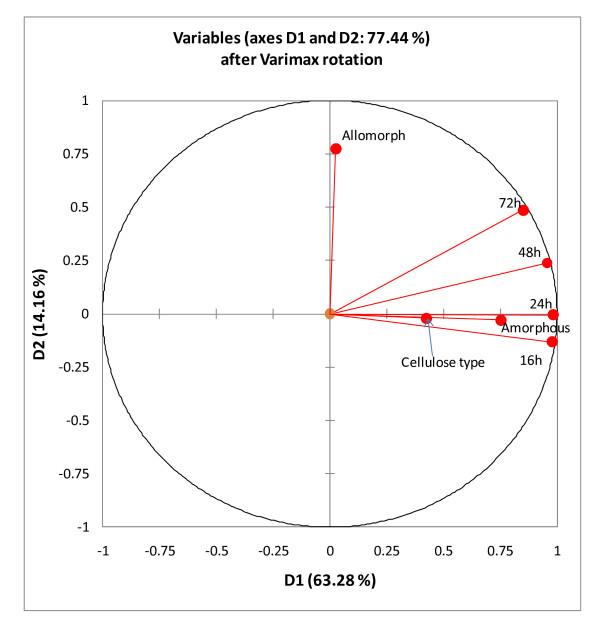
**Factor analysis of cellulose conversion levels at different digestion times (16 h to 72 h) with amorphous content, initial cellulose type, and allomorph type**. This chart shows the correlations between variables and factors after Varimax rotation.

To further elucidate the effects of crystalline structure on cellulose digestibility, factor analysis was used on the whole sample set to discover the underlying trends in the data and to determine if amorphous content or allomorph type had the most influence on digestibility. The factor analysis (Figure 8) indicated that digestibility was strongly correlated with amorphous content, particularly in the early stages of digestion. A strong correlation of cellulose conversion with allomorph type was, however, not observed, although the correlation between allomorph type and cellulose conversion improved slightly as the digestion progressed.

This work has shown that, except for corn stover, all treated samples were more digestible than the original cellulose I samples. The results of this study indicate that there is a strong correlation between amorphous content and digestibility throughout the samples tested. Under selective conditions, cellulose was converted into a more disordered, accessible and reactive form that was amenable to enzymatic hydrolysis. Sinitsyn and coworkers [38] have reported a linear relationship between the CI of cellulose samples and the specific surface area accessible to proteins. The enhanced enzymatic digestibility of the cellulose samples may therefore be attributed to a greater accessibility of the celluloses to cellulase proteins. Further studies using purified single enzymes (such as Cel7A, and others) and cellulose I, II and III_I _allomorphs prepared with comparable degrees of crystallinity will be required to elucidate the true difference in digestibility caused by the structural differences of these allomorphs.

## Conclusions

Treatment of pure celluloses with alkali and ammonia produced changes in their crystalline structures, which impacted their digestibilities when tested with a commercial enzyme complex (GC 220). Both XRD and NMR spectra show that while different allomorphs were successfully prepared, the treatments resulted in the formation of materials with differing levels of crystallinity. Treatment with NaOH converted cellulose I to cellulose II, but also produced much less crystalline cellulose. Treatment with liquid ammonia produced the cellulose III_I _allomorph; however, crystallinity depended on treatment conditions. Treatment at a final temperature of 25°C resulted in a less crystalline product, whereas treatment at 130°C or 140°C gave a more crystalline product. Treatment with NaOH produced the most digestible cellulose, followed by treatment with liquid ammonia at low temperature. The starting cellulose I samples were the least digestible (except for corn stover cellulose, which had a high amorphous content). The cellulose III_I _samples produced at higher temperatures had comparable crystallinities to the initial cellulose I samples, but achieved higher levels of cellulose conversion, particularly at longer digestion times. This is in agreement with earlier findings that the cellulose III_I _allomorph is more digestible than cellulose I. Factor analysis indicated that initial rates of digestion (up to 24 h) were most strongly correlated with amorphous content. Correlation of allomorph type with digestibility was weak but was strongest with cellulose conversion at later times.

Earlier studies have focused on determining which cellulose allomorph is the most digestible. In this study we have found that the chemical treatments to produce different allomorphs can also change the crystallinity of the cellulose, which has a significant effect on the digestibility of the substrate. When determining the relative digestibilities of different cellulose allomorphs it is essential to also consider the relative crystallinities of the celluloses being tested.

## Methods

### Materials

In this study, aqueous NaOH and liquid ammonia treatments were conducted on four celluloses: Avicel PH-101 (Sigma-Aldrich, St. Louis, MO, cat. no. 11365), α-cellulose (Sigma-Aldrich, cat. no. C8002), cotton linters (International Fiber Corporation, North Tonawanda, NY; cat. no. C10CL) and cellulose extracted from corn stover (obtained by the method described in the section on the preparation of cellulose from corn stover). These cellulose samples were selected because of their different structural properties such as DP and CI. The DP and CI of the celluloses used in this study are given in Table 5. The lower CI of the α-cellulose and corn stover cellulose is at least partially due to the lower cellulose contents of these samples, because of their contamination with xylan and lignin (Table 2). Liquid ammonia was purchased from General Air, Denver, CO. The amorphous cellulose used in this work for CI measurement using XRD was prepared from Wiley milled filter paper (Whatman No. 1) using a slight modification of the procedure developed by Schroeder and coworkers [43] described elsewhere [44]. Briefly, cellulose was heated (125°C) with paraformaldehyde in dimethyl sulfoxide (DMSO) so that the hydroxyl groups in cellulose reacted with formaldehyde and became methylolated, causing the cellulose to dissolve in DMSO. The methylol groups were removed and the cellulose regenerated by slowly adding the DMSO solution to a 0.2 M solution of sodium methoxide in methanol and 2-propanol (1:1 by volume). The amorphous cellulose precipitated and was then thoroughly washed, first with an alkoxide solution in methanol, followed by 0.1 M hydrogen chloride, and then deionized water.

### Preparation of cellulose from corn stover

Cellulose was extracted from corn stover harvested in Kearney, NE. The feedstock was made from corn stover internodes that were cut into approximately 2 cm long pieces and then cut in half length-ways. The corn stover cellulose was prepared by organosolv pretreatment using conditions similar to those previously described [45]. The corn stover internodes were pretreated at 130°C, for 56 min, using 0.57 weight percent sulfuric acid. The pretreatment resulted in hydrolysis of a majority of the xylan and solubilization of a majority of the lignin. The yield of solid after pretreatment was 53 weight percent. After pretreatment, the solid residue was washed exhaustively with water and then pressed to approximately 25% solids.

Further delignification was achieved by treatment of the solid residue with acid chlorite according to the method of Saarnio *et al*. [46]. Sodium chlorite (0.67 g per gram of biomass) was mixed with the pretreated biomass in a large zip-top plastic bag at a 10% consistency in water. After thorough wetting of the biomass, acetic acid was added at a concentration of 1 mL glacial acetic acid/per gram of biomass. The bag containing the bleaching mixture was sealed and placed in a 60°C water bath for 2 h with regular mixing. After the chlorine dioxide gas was generated it was periodically vented from the bag.

After acid chlorite delignification, the mixture was filtered on a Buchner funnel and washed with deionized water until all traces of the bleaching mixture had been removed. Water was pressed from the filter cake to an approximately 25% solids content.

### Liquid ammonia treatment

**Figure 9:**
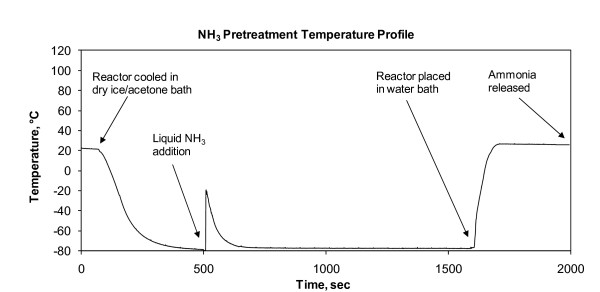
**Temperature profile for the liquid ammonia treatment conducted at 25°C for 5 min**.

The conventional method of preparing cellulose III from cellulose I was used based upon the methods described by Barry *et al*. [21] and Isogai *et al*. [47]. About 3 g to 5 g of cellulose (oven dry equivalent weight) was placed in a stainless steel reaction vessel (Model 4714, Parr Instrument Co., Moline, IL). The reaction vessel was clamped shut, weighed, and then chilled in a dry ice/acetone bath (-75°C) to facilitate transfer of liquid ammonia into the reactor at atmospheric pressure. Anhydrous liquid ammonia was added slowly to the reaction vessel in the ratio of 2 g to 3 g per gram of cellulose using a stainless steel transfer tube from a liquid ammonia cylinder equipped with an eductor tube. After adding ammonia, the vessel was immediately weighed and then cooled in the dry ice/acetone bath for 15 min. For a -33°C treatment, the vessel was opened after 90 min treatment at -75°C and allowed to vent into a hood overnight until all the ammonia had evaporated. For a final treatment temperature of 25°C, the cellulose was initially treated at -75°C for 15 min, and then the vessel was immersed in a water bath maintained at 25°C for a reaction period of either 5 min or 90 min. After completion of the desired reaction period and prior to its removal from the water bath, the treatment was terminated by immediately depressurizing the vessel in a ventilated hood. The ammonia treated cellulose was removed from the vessel and left in the hood overnight until all ammonia had evaporated. A temperature profile for the liquid ammonia treatment conducted at 25°C is shown in Figure 9. For treatment at elevated temperatures, in other words 75°C to 140°C, the cellulose was initially treated at -75°C for 15 min then the vessel was maintained at 25°C for 30 min prior to subjecting the vessel to a final heat treatment of 1 h in a preheated fluidized sand bath (Techne Inc., Burlington, NJ) maintained at the desired reaction temperature (75°C, 105°C, 130°C or 140°C). After concluding the final heat treatment and prior to its removal from the sand bath, the reaction vessel was depressurized by allowing the ammonia to leak out in a ventilated hood. After releasing the ammonia the vessel was cooled in a water bath maintained at 25°C. The ammonia treated cellulose was removed from the vessel and left in the hood overnight until all the ammonia had evaporated.

### Aqueous NaOH treatment

The aqueous NaOH treatment of cellulose samples was carried out according to the method described by Fengel *et al*. [29]. Powdered cellulose samples were soaked and stirred in 16.5 weight percent NaOH solution (2.142 g cellulose plus 250 mL NaOH) at 25°C for 2 h with air displaced by nitrogen. The NaOH solution was filtered from the sample and then the filter cake was extensively washed with distilled water until the wash was neutral. The washed solid was then freeze-dried. The aqueous NaOH treatment of all four cellulose samples was conducted with 16.5 weight percent aqueous NaOH because treatment at this concentration has been reported to cause complete conversion of cellulose I to II [29].

### Degree of polymerization measurements

The distribution of molecular weight (or DP) of cellulose samples was characterized by size exclusion chromatography (SEC). The procedure was modified from prior published methods [48-50]. To measure the molecular weight distribution of cellulose, it was necessary to dissolve the cellulose in the SEC eluent, tetrahydrofuran (THF), which was achieved by forming the tricarbanilate derivative of the celluloses.

Cellulose carbanilation was achieved by the following procedure. Cellulose samples (10 mg) that had been dried overnight at 40°C in a vacuum oven were placed in 5 mL Reactivials (Pierce, Rockford, IL), and dry pyridine (2 mL) plus phenyl isocyanate (0.4 mL) were added. Phenyl isocyanate was present in a 20-fold molar excess relative to the available hydroxyl groups in the celluloses. The reaction vials were heated to 70°C and stirred vigorously in a Pierce Reacti-Therm Heating/Stirring Module for 24 h. Reactions were quenched by adding 0.4 mL of methanol to the mixture to react with the excess phenyl isocyanate. The derivatives were precipitated in 26 mL of methanol and water (7:3, v/v) and then washed two times in the methanol and water mixture. The cellulose carbanilates were then dissolved in THF (20 mL). The solutions were filtered through 0.45 μm porosity Nylaflo (PALL, Port Washington, NY) filters prior to analysis. SEC was performed at room temperature using five columns (PLgel 10 μm 10^3^, 10^4^, 10^5^, and 10^6 ^Å, plus a larger 10^7 ^Å Shodex column) to cover the broad molecular weight range of the carbanilated celluloses. Polystyrene standards were used to calibrate retention time for molecular weight. HPLC grade THF was used as eluent at a flow rate of 1.0 mL/min. Chromatograms were obtained by monitoring the signal from a diode-array detector at a wavelength of 235 nm with a bandwidth of 10 nm. To calculate cellulose DP, molecular weights were divided by 519, the molecular weight of a repeating unit of carbanilated cellulose with the degree of substitution of 3.0.

### Enzymatic digestion

Enzymatic digestions were performed on the untreated, aqueous NaOH and liquid ammonia treated cellulose samples in duplicates. Enzymatic digestions were performed in 125 ml Erlenmeyer shake flasks at 1% solids loading at 40°C, and 130 rpm for 120 h according to National Renewable Energy Laboratory (NREL) procedure NREL/TP-510-42629 [51]. Genencor GC220 (Lot 4900759448; 121 FPU/mL, 202 mg protein/mL) cellulase enzyme preparation was added at the level of 20 mg protein per gram of cellulose (corresponding to about 12 filter paper units/g of cellulose). No additional ß-glucosidase or Multifect Xylanase was added. The total volume of the saccharification slurries after adding the enzyme and 50 mM citrate buffer (pH 4.8) was 50 mL. To determine the progress of cellulose conversion, a 2 mL aliquot of the well mixed slurries was taken at predetermined time points starting with 4 h, 16 h, and 24 h. Thereafter samples were removed every 24 h until 120 h. The samples taken were immediately filtered through PALL Acrodisc Nylon (PALL) 0.2 μm syringe filters and then refrigerated until subjected to glucose analysis. The glucose and cellobiose yields were measured by HPLC (Agilent 1100 series) using a Shodex sugar SP0810 column maintained at 85°C according to the NREL procedure NREL/TP-510-42623 [52]. The mobile phase was 0.2 μm filtered nanopure water at a flow rate of 0.6 mL/min. The sample injection volume was 10 μL and the run time 23 min. Cellulose conversion was calculated by adding the total glucose and cellobiose yields (both glucose and cellobiose were converted to glucan equivalent) for each hydrolysis time point.

### X-ray diffraction

XRD was performed with a Scintag PTS four-circle goniometer (Scintag Inc., Cupertino, CA) using CuKα radiation (0.15406 nm) generated at 45 kV and 36 mA. The source slits were 2.0 mm and 4.0 mm at a 290 mm goniometer radius and the detector slits were 1.0 mm and 0.5 mm at the same radius. Dried cellulose samples were mounted onto a quartz substrate using a few drops of diluted glue. This diluted glue is amorphous when dry and adds almost no background signal. Scans were obtained from 8 to 42 degrees two-theta in 0.05 degree steps. The CI of cellulose was calculated from the XRD spectra according to the amorphous subtraction method described elsewhere [44]. Briefly, a diffractogram of amorphous cellulose was subtracted from the diffractograms of the test samples so as to remove the influence of the amorphous component in the diffractograms. Then the ratio of the integrated areas of the subtracted diffractogram to the original diffractogram was calculated to give the CI of the sample. Scherrer's equation [31,32] was used for estimating crystallite size:

$$\beta =\frac{k\lambda }{\tau \cos\theta }$$

where *λ *is the wavelength of the incident X-ray (1.5418 Å), *θ *the Bragg angle corresponding to the (002) plane, *β *the full-width at half maximum (FWHM) of the X-ray peak corresponding to the (002) plane, *τ *is the X-ray crystallite size, and *k *is a constant with a value of 0.89 [25,53].

### ^13^C CPMAS solid-state NMR analysis

The high-resolution ^13^C CPMAS NMR spectra were recorded on a Bruker Avance 200 MHz spectrometer (Bruker BioSpin Corporation, Billerica, MA) with a 7 mm probe, operating at 50.13 MHz for ^13^C, at room temperature. The spinning speed was 7000 Hz, contact pulse 2 ms, acquisition time 51.3 ms and delay between pulses 4 s, with 20000 scans. The adamantane peak was used as an external reference (δ_C _38.3 ppm). The samples were hydrated with deionized water (around 43%) before recording the spectra. The ^13^C chemical shifts were given in δ values (ppm) and each peak in the cellulose I, II and III_I _spectra were assigned according to literature values [35,36,54,55]. Cellulose I: C6-amorphous (61.3 to 62.1 ppm), C6-crystalline (65.2 to 65.8 ppm), C2, C3 and C5 (71.2 to 75.6 ppm), C4-amorphous (83.5 to 84.4 ppm), C4-crystalline (88.2 to 89.6 ppm), C1 (104.2 to 107.8 ppm). Cellulose II: C6 (61.4, 62.2, 62.7 ppm), C2, C3 and C5 (72.6, 72.9, 73.7, 74.4, 74.6, 75.9, 76.2, 76.5 ppm), C4 (84.2, 84.4, 86.1, 87.4, 88.3, 88.6 ppm), C1 (104.8, 106.9 ppm). Cellulose III_I_: C6 (62.4 ppm), C5 (72.9 ppm), C2 (73.5 ppm), C3 (75.9 ppm), C4 (87.9 ppm), C1 (104.9 ppm). The CI was determined by applying the amorphous subtraction method to the NMR spectra [44].

### Factor Analysis

Principal factor analysis was conducted on the cellulose conversion data obtained after 16 h, 24 h, 48 h and 72 h digestion, combined with the amorphous content data and the categorical variables cellulose type and allomorph type. The categorical variable cellulose type was coded (-2 = avicel; -1 = α-cellulose; 1 = cotton linters; 2 = corn stover) as was the allomorph variable (-1 = cellulose I; 0 = cellulose II; 1 = cellulose III). The factor analysis was performed with Pearson correlation and Varimax rotation using XLSTAT (Addinsoft USA, New York, NY), an add-in program to Microsoft Excel 2007 (Microsoft Corporation, Redmond, WA).

## Competing interests

The authors declare that they have no competing interests.

## Authors' contributions

AM prepared all of the cellulose II and III_I _samples used in this study, obtained X-ray powder diffraction spectra on the various cellulose samples, calculated CI values from all XRD spectra, measured the enzymatic digestibilities of the samples, and drafted the manuscript. RK obtained the solid state ^13^C NMR spectra of the samples, calculated the CI data from the NMR spectra, and drafted sections of the manuscript related to the NMR analyses. MEH helped conceive the study and revise the manuscript. DKJ contributed to the original conception of the study, advised on the design and progress of the experimentation, performed the DP and factor analyses, and helped draft and revise the manuscript. All authors critically revised the draft and approved the final manuscript.
